# Refractory migraine in a headache clinic population

**DOI:** 10.1186/1471-2377-11-94

**Published:** 2011-08-01

**Authors:** Pablo Irimia, Jose-Alberto Palma, Roberto Fernandez-Torron, Eduardo Martinez-Vila

**Affiliations:** 1 Department of Neurology. Headache Unit. University Clinic of Navarra. Av. Pio XII, 36, Pamplona 31008, Spain

## Abstract

**Background:**

Many migraineurs who seek care in headache clinics are refractory to treatment, despite advances in headache therapies. Epidemiology is poorly characterized, because diagnostic criteria for refractory migraine were not available until recently. We aimed to determine the frequency of refractory migraine in patients attended in the Headache Unit in a tertiary care center, according to recently proposed criteria.

**Methods:**

The study population consisted of a consecutive sample of 370 patients (60.8% females) with a mean age of 43 years (range 14-86) evaluated for the first time in our headache unit over a one-year period (between October 2008 and October 2009). We recorded information on clinical features, previous treatments, Migraine Disability Assessment Score (MIDAS), and final diagnosis.

**Results:**

Overall migraine and tension-type headache were found in 46.4% and 20.5% of patients, respectively. Refractory migraine was found in 5.1% of patients. In refractory migraineurs, the mean MIDAS score was 96, and 36.8% were medication-overusers.

**Conclusions:**

Refractory migraine is a relatively common and very disabling condition between the patients attended in a headache unit. The proposed operational criteria may be useful in identifying those patients who require care in headache units, the selection of candidates for combinations of prophylactic drugs or invasive treatments such as neurostimulation, but also to facilitate clinical studies in this patient group.

## Background

Migraine is a common and disabling primary headache disorder [1-3]. Despite substantial advances in migraine therapy [4], many patients are considered refractory to treatment. Although the term refractory migraine (RM) has been used in the literature for decades, operational criteria were not defined until recently [5,6].

The Refractory Headache Special Interest Section of the American Headache Society proposed the criteria for both RM and refractory chronic migraine (R-CM) [6,7]. According to this definition, refractory migraineurs must meet the International Classification of Headache Disorders, Second Edition (ICHD-2) criteria for migraine or chronic migraine [1]. Headaches need to cause significant interference with function or quality of life despite modification of triggers, lifestyle factors, and adequate trials of acute and preventive medicines. The trials with acute medicines should include both a triptan and dihydroergotamine (DHE) intranasal or injectable formulation and either nonsteroidal anti-inflammatory drugs (NSAID) or combination analgesics. The definition requires that patients fail adequate trials of preventive medicines with established efficacy, alone or in combination, from more than 2 of 4 drug classes including: beta-blockers, anticonvulsants, tricyclics, and calcium channel blockers. The definition also includes modifiers for the presence or absence of medication overuse headache (MOH), and with or without significant disability, according to the Migraine Disability Assessment Score (MIDAS).

The proportion of patients with RM and R-CM attending headache units seems to be growing [8], but the actual epidemiology of this disorder is unknown [9].

The purpose of this study was to investigate the frequency of refractory migraineurs, according to the proposed criteria for episodic RM and R-CM [6,7] among the patients who seek care for the first time in a headache unit.

## Methods

### Study Population

This study was based on a sample of consecutive patients attended for the first time (only new patients) in the headache unit based in the Department of Neurology at the University Clinic of Navarra, during a one-year period (from October 2008 to October 2009). Patients attended in our headache unit may be referred from primary care, the emergency department, other health professionals, or self-referred. All patients underwent a clinical interview and neurological examination by the first author. Information on clinical features, possible triggers, previous or current preventive and abortive treatments and diagnosis were recorded. Additional investigations to rule out secondary causes of headache (mainly cerebral magnetic resonance imaging) were performed depending on the results of the clinical history and neurological examination. MIDAS was used as the clinical measure of headache-related disability [10]. MIDAS scores can be classified into four severity grades: little or none (0-5), mild (6-10), moderate (11-20), and severe (≥ 21). A standardized questionnaire based on the ICHD-2 was used for new patients attended in the headache unit. The information obtained from the patients was stored in a Headache database for statistical analysis. Each patient or the patient's guardian gave their informed consent and the local Ethics Committee approved the study.

### Classification

Headaches were classified according to the diagnostic criteria of the International Classification of Headache Disorders, second edition (ICHD-2) [1] and revised criteria for chronic migraine and medication overuse headache (MOH) [11]. To define patients with episodic RM and R-CM, we used the criteria proposed by Schulman et al. [6], which were modified during the validation procedure [7].

### Statistics

Statistical analyses were performed using SPSS version 15.0 for Windows (SPSS Inc, Chicago, IL, USA). For categorical (qualitative) data, the χ^2 ^test was used to check the differences between the two groups. When the expected frequencies were less than 5, Fisher's exact test was performed.

For comparisons of two or more means, the analysis of variance (ANOVA) was performed. When a significant result was obtained in the ANOVA test, a *post-hoc *analysis (Scheffé's test) was carried out to perform multiple comparisons.

In all cases, statistical significance was defined as *p *< 0.05.

## Results

### Patients and diagnoses

During the study period, 370 Caucasian patients (225 females) with headache were attended for the first time (new patients) in our headache clinic. The mean age was 43 years (range: 14-86 years). The final diagnoses of the patients are specified in Table 1. Of the participants, 172 (46.4%) had migraine, 76 (20.5%) tension-type headache, 13 patients (3.5%) had cluster headache or other trigeminal autonomic cephalalgias and 22 (5.9%) had other primary headaches. In our group, 70 patients (18.9%) had secondary headaches and nine (2.4%) other headache types (including two patients with primary trochlear headache). Forty patients (10, 8%) were diagnosed as having MOH. The headache most frequently associated with MOH was migraine in 27 patients (67.5% of the cases of MOH). In relation with headache frequency at admission, 125 patients (33.7%) reported headache more than 15 days per month in the previous three months.

**Table 1:** Diagnoses according to ICHD-2 and revised ICHD-2 criteria, given at the Headache Clinic of the University Clinic of Navarra

Diagnoses	n	%
Migraine	172	46.4
Migraine without aura	110	29.7
Migraine with aura	42	11.3
Chronic migraine	20	5.4
Tension-type headache (TTH)	76	20.8
Episodic infrequent TTH	5	1.3
Episodic frequent TTH	61	16.4
Chronic TTH	10	2.7
Cluster headache and other trigeminal autonomic cephalalgias	13	3.5
Cluster headache	12	3.2
Paroxysmal hemicrania	1	0.2
Other primary headaches	22	5.9
Primary stabbing headache	7	1.9
Primary cough headache	1	0.2
Primary exertional headache	2	0.5
Primary thunderclap headache	2	0.5
Hemicrania continua	2	0.5
New daily-persistent headache	8	2.1
Secondary headaches	70	18.9
Medication-overuse headache	40	10.8
Cervicogenic headache	15	4.0
Other secondary headaches	15	4.0
Cranial neuralgias and central causes of facial pain	8	2.1
Trigeminal neuralgia	5	1.3
Occipital neuralgia	3	0.8
Other headache types	9	2.4
Total	370	100

International Classification of Headache Disorders, second edition (ICHD-2); n: number of patients; %: percentages.

### RM and R-CM

19 patients (11 women) fulfilled the diagnostic criteria for RM or R-CM, representing 5.1% of all patients. The features of RM or R-CM are summarised in Table 2. Of the 19 patients, four (one woman) met the criteria for RM, while 15 patients (10 women) met the criteria for R-CM. The mean age of refractory migraineurs was 43 years (range, 26-66 years)

**Table 2:** RM and R-CM characteristics*

*Feature*	Study group (n = 19)
Sex	
Female	11 (57.9%)
Male	8 (42.1%)
	
Age, mean (range)	43 (26-66)
	
Age at headache onset, mean (range)	22.7 (13-36)
	
Type of migraine	
Episodic migraine	4 (21.1%)
Chronic migraine	15 (78.9%)
	
MIDAS score, mean (range)	96 (45-180)
	
Medication overuse	
Yes	7 (36.8%)
No	12 (63.2%)

*Values are number (percentage) unless otherwise stated

All refractory migraineurs had undergone abortive treatments with triptans and NSAID, except one patient who was allergic to NSAID. Intranasal or injectable dihydroergotamine was not used in our patients because this drug is not available in Spain. All possible triggers and lifestyle factors were addressed.

All refractory migraineurs had undergone a therapeutic trial with more than 2 groups of preventive drugs for at least 2 months at optimal or maximal tolerated doses, unless terminated early due to adverse effects. 17 patients had received tricyclic antidepressants (mainly amitriptyline, but also nortriptyline), 14 patients had received beta-blockers (mainly propranolol, but also atenolol and metoprolol), 16 patients had received calcium-channel-blockers (mainly flunarizine) and 18 patients had received anticonvulsants (14 topiramate, 10 valproate, 5 gabapentin, 2 lamotrigine, 1 pregabaline, 1 carbamazepine and 1 zonisamide). Three patients experienced intolerance to preventive drugs (topiramate in 3 and amitriptyline in 1).

Additionally some patients had received other preventive treatments, with at least one positive randomized controlled trial in migraine, but not included in the current proposed criteria for RM and R-CM [6,7]. These additional treatments include botulinum toxin infiltration in 3 patients, pizotifen in 1, and vitamin B2 in 1. These treatments were not taken into account in establishing the diagnosis of RM or R-CM. Other common treatments used in refractory migraineurs to manage comorbid psychiatric disorders included serotonine reuptake inhibitors in 7 patients and lithium in 3 patients. Two patients had also received treatment with occipital nerve blocks.

The mean MIDAS score in refractory migraineurs was 96 (45-180), and therefore, in these cases the diagnosis of RM and R-CM may be qualified with the modifier "with significant disability".

In our headache clinic, 36, 8% of patients with RM or R-CM overused analgesics and therefore in these cases the diagnosis of RM and R-CM may be qualified with the modifier "with medication overuse".

For comparison purposes, refractory migraineurs were classified into four groups: RM without MOH, RM with MOH, R-CM without MOH and R-CM with MOH (Table 3). No statistical differences between the groups were found when comparing for sex, age, age of headache onset, preventive drugs and abortive medicines. Only when comparing the MIDAS scores, differences between the three groups were statistically significant (*F *= 11.37, *p *= 0.001). Sheffé's test demonstrated that differences in MIDAS scores were significant (*p *= 0.001) when comparing the groups of episodic RM with MOH (mean MIDAS of 65) or RM without MOH (mean MIDAS of 56) against the group of R-CM with MOH (mean MIDAS of 131.6). The differences in MIDAS scores were also significant (*p *= 0.02) when comparing the group of R-CM with MOH (mean MIDAS of 131.6) against the group of R-CM without MOH (mean MIDAS of 92.5).

**Table 3:** RM and R-CM subgroups characteristics

	RM	RM	R-CM	R-CM
	without MOH	with MOH	without MOH	with MOH
n	2	2	10	5
Age, mean (range)	34.5 (24-43)	38 (28-48)	42.3 (31-60)	49.8 (36-66)
MIDAS score, mean (range)	56 (50-62)	65 (50-80)	92.5 (45-105)	131.6 (94-180)

MOH: Medication Overuse Headache; n: number of patients; RM: refractory migraine; R-CM: refractory chronic migraine

## Discussion

RM and R-CM accounted for 5.1% of patients attended for the first time in our headache unit. To our knowledge, this is the first study to describe the frequency of refractory migraineurs in a headache unit with the current diagnostic criteria. The proposed criteria for both RM and R-CM introduce the new term "refractory" into the classification of chronic headache [12-14]. The ICHD-2 diagnostic criteria do not provide any usable classification for this relatively common condition between the patients attended in tertiary headache units. Refractory headache patients are usually referred to specialized clinics [15] and most of them require follow-up visits for several months. Headache specialists intuitively recognize when a patient is refractory, but with the proposed criteria for both RM and R-CM is possible to define the frequency of this patient population. The benefits of the newly proposed diagnostic criteria are not only the possibility to study the epidemiology of RM and R-CM, but also to define the treatment strategies for these challenging patients.

The overall age and sex distribution among headache patients in our headache unit is similar to previous studies in other headache units in Spain [16]. In our RM and R-CM patients, headaches caused a significant interference with function and quality of life. All our RM and R-CM patients experienced severe disability (MIDAS ≥ 21) and therefore the diagnosis of RM and R-CM may be qualified with the modifier "with significant disability". The noteworthy MIDAS found in refractory migraineurs is consistent with the scores observed in those migraneurs treated with neurostimulation after the failure of several preventive drugs [17], and may be related to the significant degree of disability caused by refractory pain. Moreover, chronic migraine is more disabling than episodic migraine in the population [3], and likewise in our sample, the highest MIDAS corresponds to R-CM. Our study shows that the proportion of patients with chronic migraine is very similar to the proportion of RM and R-CM. These findings can be explained because refractory migraineurs may suffer from episodic or chronic migraine. Furthermore, in the chronic migraine revised ICHD-2 criteria [11], medication-overuse has to be ruled out, and therefore some migraneurs with near daily headache have to be classified as having MOH. Between our patients classified as having medication-overuse headache, most were migraineurs. According to revised ICHD-2 criteria [17], if a patient has headache on ≥ 15 days/month after > 3 months of regular overuse of one or more acute/symptomatic treatment drugs, and the headache has developed or markedly worsened during medication overuse, then a diagnosis of MOH can be made. If the headache persists after 2 months of withdrawal, then a new diagnosis of chronic migraine is given. Considering that definition of RM and R-CM was to have worldwide application, it was not considered practical to avoid medication overuse. For that reason, medication overuse was considered a modifier, although, ideally, medications potentially causing MOH must be withdrawn before a patient with migraine can be identified as refractory.

Our study has some evident limitations. The best approach to estimate the proportion of patients with RM and R-CM is a population based study and not a single center study, such as the present one. However, the information regarding the failure of adequate trials of acute treatments and preventive drugs (required for the diagnosis of RM and R-CM) is difficult to obtain by means of questionnaires or phone surveys, which are the usual methods in population-based studies. This sort of information is best obtained in person by an experienced neurologist with enough time to obtain the history, and review the clinical records, as occurs in most of headache units. Also, our data were obtained from a single headache unit and therefore our findings may not be representative of other tertiary headache centres. However, the proportion of patients with chronic headache and the distribution of diagnoses observed in the present study are similar to other headache units. In our sample, and also in several series of patients attended in headache units, patients with chronic headaches were over-represented [18]. Concerning headache diagnoses, the high frequency of tension-type headache observed in our population, but also in previous reports [16,19,20], contrasts with the experience of other headache units [21,22]. These differences may be partially explained by the pattern of referral to the headache unit (some of our patients are self-referred) and the application of ICHD-II criteria in the present study. In fact, the distribution of diagnoses in the present work is similar to headache units in which patients were classified according to ICHD-2 [16,23]. Recall bias may constitute an additional limitation and may have affected our results. Patients might have had problems remembering the preventive therapeutic groups tried, or whether the trial was adequate, which is needed for the definition of RM and R-CM. However, preventive drugs were used during prolonged periods of time and most of the patients remembered details of the trials; in the remaining cases, such information was obtained from medical records.

The reliability and external validity of the proposed diagnostic criteria for RM need to be established in future studies. We consider that the present criteria are valid and exhaustive, but there are two shortcomings. First, the use of ergotics, even in oral presentation, in patients with migraine is not common [24]. In Spain and other countries, DHE is not available either intranasal or injectable forms. Thus, our patients do not meet the criterion that a trial with DHE (intranasal or injectable formulation) is needed for RM or R-CM diagnosis. Given that the definitions of RM and R-CM are going to be used worldwide, and the unavailability intranasal or injectable DHE in several countries, this criterion must be reviewed. In addition, MOH is included in the proposed criteria as a modifier and patients can be classified as having R-CM with or without MOH. We agree that diagnoses of RM and R-CM may be made even in patients who overuse analgesics. However, we would specify in the criteria that overused medication needs to be withdrawn for two months on at least one occasion, before the patient can be classified as refractory.

## Conclusion

In conclusion, RM and R-CM are relatively common conditions among patients evaluated for the first time in a headache unit. Patients with RM and R-CM are severely disabled and need to be cared by neurologists attached to specialized headache clinics. The proposed operational criteria may be useful not only to identify those patients who require care in headache units but also to select the candidates for combinations of prophylactic drugs or invasive treatments, such as neurostimulation, and to facilitate clinical studies in this patient group.

## List Of Abbreviations

(RM): Refractory migraine; (MIDAS): Migraine Disability Assessment Score; (ICHD-2): International Classification of Headache Disorders, second edition; (NSAID): Non-Steroidal Anti-Inflammatory Drugs; (R-CM): Refractory chronic migraine; (MOH): Medication overuse headache.

## Competing interests

The authors declare that they have no competing interests.

## Authors' contributions

**PI**: involved in the conception and design, analysis and interpretation of data, drafting the manuscript and revision for important intellectual content and has read and approved the final manuscript. **JAP**: involved in acquisition of data, analysis and interpretation of data, drafting the manuscript, revision for important intellectual content and has read and approved the final manuscript. **RFT**: involved in acquisition of data, revision for important intellectual content and has read and approved the final manuscript. **EMV**: involved in the conception and design, and revision for important intellectual content and has read and approved the final manuscript.

## Funding

None received.

## Pre-publication history

The pre-publication history for this paper can be accessed here:

http://www.biomedcentral.com/1471-2377/11/94/prepub
